# Nasal CPAP in the Pediatric Ward to Reduce PICU Admissions for Severe Bronchiolitis?

**DOI:** 10.3390/pediatric15040055

**Published:** 2023-10-13

**Authors:** Melodie O. Aricò, Diana Wrona, Giovanni Lavezzo, Enrico Valletta

**Affiliations:** 1 Department of Pediatrics, G.B. Morgagni—L. Pierantoni Hospital, AUSL Romagna, 48018 Forli, Italy; melodieolivialoredanarosa.arico@auslromagna.it; 2 School of Specialization in Pediatrics, University of Bologna, 40138 Bologna, Italy; diana.wrona@studio.unibo.it (D.W.); giovanni.lavezzo@studio.unibo.it (G.L.)

**Keywords:** bronchiolitis respiratory syncytial virus, high-flow nasal cannula, continuous positive air pressure, oxygen administration, pediatric ward, pediatric intensive care unit

## Abstract

In treating acute bronchiolitis in infants, the decision to use continuous positive airway pressure (CPAP) often involves infant referral from the pediatric ward to the pediatric intensive care unit (PICU). We present our experience of CPAP use in a general pediatric ward, aiming to reduce the pressure on the PICU in recent outbreaks of bronchiolitis. Clinical data of patients less than 12 months of age and admitted for bronchiolitis from 1 October 2021 to 31 March 2023 were retrospectively collected. Of 82 infants admitted for bronchiolitis, 16 (19%) were treated with nasal CPAP (nCPAP group); of the remaining 66, 21 (26%) were treated with a low-flow nasal cannula (LFNC) only, 1 (1%) was also treated a with high-flow nasal cannula (HFNC), 12 (15%) were treated with an HFNC only, and 41 (50%) were treated without oxygen support (no-nCPAP group). Overall, coinfection with RSV and SARS-CoV-2 was observed in three patients and SARS-CoV-2 infection was observed in two patients. None of them required any type of oxygen support. Only 3/16 (19%) infants in the nCPAP group were referred to the PICU due to worsening clinical conditions despite nCPAP support. In our experience of treating epidemic bronchiolitis, nCPAP can be safely managed in a general pediatric ward, thus reducing the burden of admissions to the PICU. Training and regular updating of the pediatric staff, careful monitoring of the patient, and close cooperation with the PICU were instrumental for our team.

## 1. Introduction

Respiratory syncytial virus (RSV) epidemics typically follow a seasonal pattern, peaking in December and January [1]. The COVID-19 pandemic disrupted RSV seasonality, and recent, post-pandemic epidemics of bronchiolitis have raised serious clinical and organizational problems in many regions of the world [2]. In the autumn and winter seasons of 2021–2022 and 2022–2023, an anticipation of the epidemic peaks of infection and an increased case number have been observed, probably secondary to the “immunological debt” due to public health measures aimed at controlling the spread of SARS-CoV-2 [3,4,5,6,7,8].

International recommendations have enhanced the awareness of managing increased patient loads, especially in pediatric hospitals and intensive care units [9,10,11]. Indeed, the incidence of RSV in infants was higher than in previous years, suggesting a wider viral spread.

The main concern was regarding severe bronchiolitis due to RSV or other viral agents, which could have required an increased number of hospitalizations in a small amount of time with an enhanced burden for pediatric wards and pediatric intensive care units (PICU) [7]. In Korea, among hospitalized children for RSV bronchiolitis, the rate of those requiring respiratory support was significantly increased in the 2021–2022 season (32.5%) as compared with 2019–2020 (19.4%) [12]. Admissions for RSV infection tripled in New Zealand in the 2021 season both in pediatric wards and in PICUs [13]. PICU admissions were also increased in the USA [14]. In the same period, in Italy, a significant increase (34% vs. 19%) in red and yellow code assignments in emergency departments for bronchiolitis was registered in comparison with previous years. Some authors describe a tripled need for referrals to PICUs with the same rate of admissions [5]. According to other authors, the post-COVID-19 period (2021–2022) was also characterized by an increased rate of hospitalizations [4]. Overall, the recommendation was to prepare for abnormal seasonal RSV outbreaks in terms of incidence, distribution, and severity. In bronchiolitis, the assessment and monitoring of blood oxygen saturation (SO_2_) levels are of paramount importance in evaluating the severity of respiratory involvement and the advisability of hospitalization as well as the need for increasing ventilatory support during the disease [15,16,17,18,19,20]. Consequently, oxygen therapy has been recognized as the basis of treatment in bronchiolitis even if the management of bronchiolitis presents a significant variation and a univocal approach is still questioned [21].

In recent years—besides conventional, low-flow oxygen therapy with a nasal cannula (LFNC) or a mask—the use of a high-flow nasal cannula (HFNC) and noninvasive ventilation therapies like continuous positive air pressure (CPAP) have emerged as possible alternatives to tracheal intubation and conventional invasive ventilation in patients with moderate-to-severe bronchiolitis [22]. If HFNC is nowadays frequently used outside PICUs, CPAP remains a prerogative of intensive areas, and the decision to use it often involves infant referral from pediatric wards to PICUs [15,23]. Recent studies suggest that about 5% to 25% of children with bronchiolitis may require hospitalization in a PICU to receive respiratory support, mainly CPAP [5,6,24,25].

Few studies have reported on the use of CPAP in pediatric wards and, paradoxically, the availability of HFNCs outside PICUs could have sometimes resulted in an increase in PICU transfers with relative organizational costs and psychological burdens for patients and their families [23,24,25,26,27,28,29,30,31]. Recent European studies confirm that the average hospitalization costs for infants treated in PICUs for bronchiolitis are one-and-a-half to fourfold higher compared to those treated in a ward [24,29].

We used, for the first time, nasal CPAP in a pediatric ward during the 2021–2022 RSV outbreak. We decided to replicate this during the 2022–2023 epidemics to reduce the PICU requirement in an epidemiological situation that predicted an overload of patients for PICUs.

We retrospectively collected our case series discussing the main clinical and organizational issues.

## 2. Materials and Methods

Clinical data of all patients less than 12 months old and hospitalized for bronchiolitis from 1 October 2021 to 31 March 2023 were collected. No hospitalized patients for bronchiolitis were excluded. The diagnosis of bronchiolitis was made on a clinical basis in children admitted to the emergency room (ER) with compatible respiratory symptoms, namely rhinitis, wheezing, respiratory distress, and auscultatory findings of crackles and wheezing in multiple lung fields.

The hospitalization criteria were as follows: moderate-to-severe bronchiolitis, an oxygen peripheral saturation persistently below 92%, moderate-to-severe respiratory distress, episodes of apnea, a reduction in oral feeding, relevant social issues (domicile far from the hospital and reliability of the caregiver), and the presence of clinical risk factors (chronic pulmonary disease, hemodynamically significant heart disease, age less than 3 months, prematurity, neuromuscular disorder, immunodeficiency, and other significant comorbidities). The assessment of the severity of bronchiolitis was made according to international guidelines [15,16,17,18,19,20].

Patients were divided into two groups, the nCPAP group and no-nCPAP group, according to the use of nCPAP during their stay in hospital.

Low-flow O_2_ was administered with humidified O_2_ via a nasal cannula (LFNC) in patients with persistently <92% SO_2_ levels. SO_2_ was monitored throughout the entire duration of O_2_ supplementation. Treatment failure was considered if an FiO_2_ level greater than 50% was needed to maintain a SO_2_ level ≥ 92% or there was lack of improvement or a worsening of respiratory distress and vital signs were registered.

HFNC was started in patients who had moderate respiratory distress and/or respiratory acidosis (pH < 7.35, pCO_2_ > 45 mmHg) upon arterialized capillary blood gas analysis. AirvO2 equipment (Fisher and Paykel Healthcare, Inc., Auckland, New Zealand) was used with continuous cardio-oximetric monitoring. The initial flow was set at 2 L/kg/min with an FiO_2_ level of 30% and with subsequent FiO_2_ titration to maintain SatO_2_ levels between 92% and 97%. The criteria for considering treatment upgrading were a failure to improve the respiratory rate or heart rate, respiratory distress within the first 12 h of HFNC initiation, and clinical signs of a risk of respiratory exhaustion.

The nCPAP starting criteria were a failure of LFNC or HFNC oxygen support, clinical signs of a risk for respiratory exhaustion, and severe respiratory distress with or without respiratory acidosis. The devices used were a Fabian Therapy evolution SW 4.0 nasal CPAP device with a nasal interface and an Infant Flow SiPAP device (both from Vyaire Medical Inc., Mettawa, IL, USA). The starting pressure of nCPAP was 5 mBar, which was increased to up to 6.5–7 mBar with an initial FiO_2_ level at 30% titrated for SatO_2_ between 92% and 97% [23]. Patients who did not show any clinical improvement (reduction in heartrate and respiratory rate and/or better respiratory dynamics), who required an increase in FiO_2_ of up to 60% with a pressure of 7.5–8 mBar, and who showed episodes of apnea were considered for PICU admission. Once nCPAP was started, breastfeeding was encouraged for non-nutritive comfort purposes; direct contact with the caregiver (generally the mother holding the baby in her arms) was also allowed to improve adaptation.

Two-tailed Student’s *t*-test and Fisher’s exact test were used to compare the nCPAP and no-nCPAP group data with the significance level set to 0.05.

## 3. Results

Clinical data of 82 patients hospitalized for bronchiolitis were collected. Of the 82 infants admitted for bronchiolitis, 16 (19%) were treated with nCPAP (nCPAP group); of the remaining 66, 21 (26%) were treated with a low-flow nasal cannula (LFNC) only, 1 (1%) was also treated with a high-flow nasal cannula (HFNC), 12 (15%) were treated with an HFNC only, and 41 (50%) were treated without oxygen support (no-nCPAP group).

The main clinical data of the two groups are summarized in Table 1.

The groups were comparable in terms of age, the presence of comorbidities, and the prevalence of RSV and SARS-CoV-2 infection. The nCPAP group had a significantly lower mean weight (*p* = 0.043) and underwent chest radiography more frequently (*p* < 0.001).

Upon admission to hospital (T_0_), the infants in the nCPAP group were more frequently dyspneic (*p* < 0.001) and tachycardic (*p* = 0.0024), and they had worse hemogasanalysis results (i.e., respiratory acidosis, hypoxemia, and hypercapnia). The progression of O_2_ and respiratory support in both groups is summarized in Table 2.

When admitted, all the patients in the nCPAP group received some kind of respiratory support (LFNC, HFNC, or nCPAP), while only 27/66 (41%) infants in the no-nCPAP group were supported with an LFNC or HFNC.

An HFNC was the first respiratory support in eight (50%) of the children in the nCPAP group and in twelve (18%) of those in the no-nCPAP group (*p* = 0.019). One infant in the nCPAP group received nCPAP as the first respiratory support, seven (44%) were treated with an LFNC as the first support, and eight (50%) received a first HFNC. As the second respiratory support, nine patients received nCPAP and six patients received an HFNC. nCPAP was the third respiratory support in six patients. The total length of support was slightly longer in the nCPAP group than in the no-nCPAP group (*p* = 0.07), while the total stay in the pediatric ward was significantly longer in the nCPAP group (*p* < 0.001). Due to worsening clinical conditions despite support with nCPAP, 3/16 (18.7%) infants were referred to the PICU after 1, 2, and 4 days of stay in the pediatric ward, respectively.

In the nCPAP group, two patients were late preterm and one was affected by an atrioventricular canal defect in chromosomopathy.

About three children who required to be referred to PICU, the first patient was a 29 days-old, born late preterm at 36 + 2; who received first HFNC than nCPAP support, arrived in PICU he continued CPAP support. The second patient was a 20 days-old neonate with-out risk factor: the patient received first HFNC than nCPAP.When he arrived in the PICU, he received CPAP support. The third patient was 7 months old with an atrioventricular canal defect in chromosomopathy who first received an LFNC and then nCPAP. After being transferred to the PICU, the patient required invasive support and later required extra-corporeal membrane oxygenation. The first and second patients were discharged after being in good clinical condition for some days, and the third patient died two months later.

Of the eighty-two patients considered, thirty-two (39%) received glucocorticoids, forty-eight (58%) received inhaled therapy (salbutamol, adrenalin), and twenty-two (27%) received at least one dose of antibiotic therapy. None of them received antiviral therapy. In the nCPAP group, three (19%) patients received antibiotic therapy, four (25%) received glucocorticoids, and three (19%) received inhaled therapy.

Overall, coinfection with RSV and SARS-CoV-2 was observed in the patients, and SARS-CoV-2 infection was observed in two patients. None of them required any type of oxygen or ventilatory support.

## 4. Discussion

The bronchiolitis epidemics after SARS-CoV-2 pandemic were characterized by an anticipation of the usual autumn–winter spread and by increased numbers of ED admissions and a high burden of hospitalization in pediatric departments [25]. In the autumn and winter seasons of 2021–2022 and 2022–2023, an anticipation of the epidemic peaks of infection and an increased case number were observed, probably secondary to the “immunological debt” due to the public health measures aimed to control the spread of SARS-CoV-2 [3,4,5,6,7,8,32,33,34]. The perspective, reiterated by media and scientific societies, of an uncontrolled increase in admissions for bronchiolitis in PICUs led us to implement, when needed, respiratory support with nCPAP in the general pediatric ward [9,10,11].

The approach to respiratory support during hospitalization was flexible and guided by the evolution of the disease. In the nCPAP group, the administration of an LFNC or HFNC was the first form of support in 94% of the patients. Oxygen administration is recognized as the mainstay of treatment for bronchiolitis with a high level of quality and a high strength of available evidence [15,16,17,18,19,20]. O_2_ administration with nasal cannulas or a mask is the most widespread and used support both in EDs and in pediatric wards. In the last decades, the use of HFNCs has gained space in the therapeutic practice of many hospital wards. In moderate–severe bronchiolitis, heated and humidified O_2_ at higher flows determines a modest positive pressure that could help to improve the patency of the upper airways, increase CO_2_ washout, and may reduce respiratory distress, even if the exact physiological mechanisms of HFNCs are not completely clear [35].

The use of different supports in bronchiolitis is still debated; HFNCs present the advantages of better tolerance and minor incidences of skin lesions and abdominal distensions compared to CPAP. On the other hand, CPAP seems to be more effective than HFNCs in moderate–severe bronchiolitis in treating hypoxemia and respiratory effort, even if this is not widely shared in the literature [35,36]. The absence of standard starting and weaning protocols when using HFNC support makes it difficult to demonstrate the real effects of this device and to compare its efficacy with CPAP.

In our experience, an HFNC was the first respiratory support—after initial stabilization with an LFNC in the ED—used in 44% of the patients in the nCPAP group and was the second measure during hospitalization in the other eight (50%) children. In both situations, the switch to using an HFNC was decided upon after an unsatisfactory correction of hypoxemia with conventional O_2_ administration and a progressive worsening of respiratory distress were observed. Despite their increasing use in treating bronchiolitis, the definitive role of HFNCs is still debated with regard to their efficacy in reducing the duration of O_2_ administration and the length of hospitalization and in preventing clinical worsening and the need for more intensive care [37,38,39,40,41,42,43,44,45]. Currently, HFNCs should be reserved for cases of failure of conventional O_2_ therapy [23,46].

Before starting to use nCPAP in the pediatric department, all the patients who did not clinically improve with an HFNC were transferred to the pediatric intensive care unit. In our experience, the use of nCPAP in the pediatric ward allowed us to avoid about 80% of PICU transfers for bronchiolitis; only 3/16 (20%) of the patients in the nCPAP group were transferred to the PICU. Our medical and nursing staff received specific training and participated in periodic refresher sessions that focused on the use of the equipment and patient monitoring. It was also envisaged that the nurse–patient ratio, usually one nurse for every six patients, could be increased to one nurse for every four patients, according to the variable needs for assistance in the presence of children receiving CPAP support. Communication with the reference PICU—located at about 20 km distance—was kept constant in consideration of possible worsening of the patients’ clinical conditions.

CPAP is increasingly being used to support patients with moderate-to-severe bronchiolitis after failure of conventional oxygen therapy and HFNC [1]. The pressure generated by CPAP reduces respiratory airway resistance and prevents the development of pulmonary atelectasis, representing, in fact, an increase in non-invasive respiratory support when compared to the use of HFNCs. However, the clinical advantages of CPAP vs. HFNCs are still under evaluation, pending more definitive evidence [16,22,23,24,47]. The decision to undertake non-invasive support with CPAP coincides, in most organizational situations, with the transfer of the child to the PICU. Nonetheless, increasing experience of pediatric personnel with CPAP equipment and the progressive evolution of technologies has led to the first reports of CPAP use in pediatric wards outside intensive care units [23,24,25,26,27,30]. In 2017, a survey of 97 hospitals in England and Wales reported the common use (56.9%) of CPAP in the pediatric wards of general hospitals, while in tertiary structures, CPAP was still used preferentially in PICUs [23]. A Spanish study reports about the use of CPAP in a trained pediatric unit with a nurse–patient ratio of 1:6 [26]. In 56% of patients, CPAP treatment was started and completed in the pediatric ward, while in the remaining 44%, a transfer to the PICU was needed.

Reduced admissions to PICUs of children with bronchiolitis may have evident organizational advantages, provided that there is appropriate training of the pediatric staff and the quick availability of the PICU to take charge of therapeutic failures. On the other hand, the increasingly widespread use of HFNCs and CPAP in treating bronchiolitis would risk exacerbating the pressure on PICUs should they remain the only organizational solution devoted to the administration of this kind of respiratory support [26,29]. Furthermore, the hospitalization costs for bronchiolitis were estimated to be 60% higher in PICUs than in pediatric wards [24].

## 5. Conclusions

This observational study, albeit retrospective and on a limited case series, suggests that respiratory support for bronchiolitis—including the use of CPAP—can be safely managed in a general pediatric ward, thus substantially reducing the burden of admissions to PICUs for non-invasive respiratory support and sparing the child and their family the experience of hospitalization in an intensive care environment. This solution could be especially suitable in a general hospital that must rely on a distant PICU and on medically assisted and safe transport. Training and regular updating of pediatric staff, careful monitoring of the patient, and close cooperation with the PICU remain fundamental issues.

## Figures and Tables

**Table 1:** Clinical features of nCPAP and no-nCPAP groups.

	nCPAP Group (*n* = 16)			No-nCPAP Group (*n* = 66)				*p*
	Patient Number (%)	Mean (SD)	Range		Patient Number (%)	Mean (SD)	Range	
Age (months)		2.93 (3.0)	0–8	Age (months)		3.93 (3.15)	0–11	0.25
Weight (Kg)		5.37 (1.8)	3–8.7	Weight (Kg)		6.58 (2.15)	2.75–14	0.043
Comorbidity	5 (31)			Comorbidity	11 (17)			0.18
RSV	13 (81)			RSV	60 (91)			0.36
SARS-CoV-2	0 (0)			SARS-CoV-2	5 (7.6)			0.57
Chest X-ray	9 (56)			Chest x-ray	9 (14)			<0.001

**Table 2:** nCPAP and no-nCPAP groups at presentation (T_0_) and respiratory support during stay in the pediatric ward.

nCPAP Group (*n* = 16)				No-nCPAP Group (*n* = 66)				
	Patient Number (%)	Mean (SD)	Range		Patient Number (%)	Mean (SD)	Range	*p*
T_0_				T_0_				
Dyspnea	16 (100)			Dyspnea	38 (58)			<0.001
Feeding refusal	15 (94)			Feeding refusal	50 (76)			0.2
Respiratory rate (rpm)		68 (14)	45–96	RR (rpm)	47	54 (13)	28–100	<0.001
Heart rate (bpm)		170 (12)	152–200	HR (bpm)	43	156 (16)	120–190	0.0024
pH		7.3 (0.05)	7.2–7.37	pH	40	7.37 (0.05)	7.22–7.48	0.001
pCO_2_ (mm Hg)		52.5 (11.2)	35.9–74.1	pCO_2_ (mm Hg)	40	39.5 (6.17)	27.3–55.3	<0.001
HCO_3_ (mmol/L)		24.6 (2.41)	20.2–29.2	HCO_3_ (mmol/L)	40	23.1 (1.55)	19.2–26.7	0.0076
SatO_2_ (%)		92 (3.7)	86–98	SatO_2_ (%)	57	94.8 (4.62)	80–100	0.029
First support				First support				
LFNC	7 (44)			LFNC	15 (23)			0.12
Length (hours)		3.85 (2.19)	1–7	Length (hours)		48 (46)	3–168	
HFNC	8 (50)			HFNC	12 (18)			0.019
FiO_2_ (%)		26.5 (2.27)	25–30	FiO_2_ (%)		27 (5)	21–35	
Length (hours)		26.8 (24.6)	4–68	Length (hours)		61 (32)	1–112	
nCPAP	1 (6)							
Starting PEEP (cm H_2_O)		5						
Max PEEP (cm H_2_O)		6.5						
FiO_2_		30						
Length (hours)		120						
Second support				Second support				
HFNC	6			HFNC	4			
FiO_2_ (%)		27.5 (4.18)	25–35	FiO_2_ (%)		25 (4)	21–29	
Length (hours)		17.6 (20)	2–53	Length (hours)		64 (14)	24–73	
nCPAP	9							
Starting PEEP (cmH_2_O)		6.25 (0.53)	5.5–7					
Max PEEP (cmH_2_O)		6.6 (0.52)	5.5–7					
FiO_2_ (%)		33 (21.6)	23–90					
Length (hours)		54.7 (47.3)	5–160					
Third support								
nCPAP	6							
Starting PEEP (cm H_2_O)		6.5 (0.5)	6–7					
Max PEEP (cm H_2_O)		6.7 (0.44)	6–7					
FiO_2_ (%)		33 (21.6)	25–40					
Length (hours)		86 (57)	36–156					
Length of support in the pediatric ward (hours)		96 (55)	12–216	Length of support in the pediatric ward (hours)		70 (35)	4–168	0.07
Stay in the pediatric ward (days)		6.35 (2.7)	1–12	Stay in the pediatric ward (days)		3.95 (2.07)	1–10	<0.001

## Data Availability

No new data were created or analyzed in this study. Data sharing is not applicable to this article.

## References

[1] Rose E.B., Wheatley A., Langley G., Gerber S., Haynes A. (2018). Respiratory Syncytial Virus Seasonality—United States, 2014–2017. MMWR Morb. Mortal. Wkly. Rep..

[2] Olsen S.J., Winn A.K., Budd A.P., Prill M.M., Steel J., Midgley C.M., Kniss K., Burns E., Rowe T., Foust A. (2021). Changes in Influenza and Other Respiratory Virus Activity during the COVID-19 Pandemic—United States, 2020–2021. MMWR Morb. Mortal. Wkly. Rep..

[3] Guerrero-Del-Cueto F., Ramos-Fernandez J.M., Leiva-Gea I., Reina-Moreno E., Ortiz-Ortigosa A., Carazo-Gallego B., Cordon-Martinez A.M., Moreno-Perez D., Nuñez-Cuadros E. (2023). Bronchiolitis before and after the SARS-CoV-2 pandemic: Twelve years of experience in a Spanish paediatric hospital. Pediatr. Pulmonol..

[4] Curatola A., Graglia B., Ferretti S., Covino M., Pansini V., Eftimiadi G., Chiaretti A., Gatto A. (2023). The acute bronchiolitis rebound in children after COVID-19 restrictions: A retrospective, observational analysis. Acta Biomed..

[5] Faraguna M.C., Lepri I., Clavenna A., Bonati M., Vimercati C., Sala D., Cattoni A., Melzi M.L., Biondi A. (2023). The bronchiolitis epidemic in 2021–2022 during the SARS-CoV-2 pandemic: Experience of a third level centre in Northern Italy. Ital. J. Pediatr..

[6] Carlone G., Graziano G., Trotta D., Cafagno C., Aricò M.O., Campodipietro G., Marabini C., Lizzi M., Fornaro M., Caselli D. (2023). Bronchiolitis 2021–2022 epidemic: Multicentric analysis of the characteristics and treatment approach in 214 children from different areas in Italy. Eur. J. Pediatr..

[7] Bozzola E., Barni S., Villani A. (2022). Respiratory Syncytial Virus Pediatric Hospitalization in the COVID-19 Era. Int. J. Environ. Res. Public. Health.

[8] Munkstrup C., Lomholt F.K., Emborg H.D., Møller K.L., Krog J.S., Trebbien R., Vestergaard L.S. (2023). Early and intense epidemic of respiratory syncytial virus (RSV) in Denmark, August to December 2022. Eurosurveillance.

[9] https://www.ecdc.europa.eu/en/publications-data/intensified-circulation-respiratory-syncytial-virus-rsv-and-associated-hospital.

[10] Hamid S., Winn A., Parikh R., Jones J.M., McMorrow M., Prill M.M., Silk B.J., Scobie H.M., Hall A.J. (2023). Seasonality of Respiratory Syncytial Virus—United States, 2017–2023. MMWR Morb. Mortal. Wkly. Rep..

[11] Eden J.S., Sikazwe C., Xie R., Deng Y.M., Sullivan S.G., Michie A., Levy A., Cutmore E., Blyth C.C., Britton P.N. (2022). Off-season RSV epidemics in Australia after easing of COVID-19 restrictions. Nat. Commun..

[12] Kim Y.K., Song S.H., Ahn B., Lee J.K., Choi J.H., Choi S.H., Yun K.W., Choi E.H. (2022). Shift in Clinical Epidemiology of Human Parainfluenza Virus Type 3 and Respiratory Syncytial Virus B Infections in Korean Children before and during the COVID-19 Pandemic: A Multicenter Retrospective Study. J. Korean Med. Sci..

[13] Hatter L., Eathorne A., Hills T., Bruce P., Beasley R. (2021). Respiratory syncytial virus: Paying the immunity debt with interest. Lancet Child Adolesc. Health.

[14] Movva N., Suh M., Reichert H., Hintze B., Sendak M.P., Wolf Z., Carr S., Kaminski T., White M., Fisher K. (2022). Respiratory Syncytial Virus during the COVID-19 Pandemic Compared to Historic Levels: A Retrospective Cohort Study of a Health System. J. Infect. Dis..

[15] Manti S., Staiano A., Orfeo L., Midulla F., Marseglia G.L., Ghizzi C., Zampogna S., Carnielli V.P., Favilli S., Ruggieri M. (2023). UPDATE—2022 Italian guidelines on the management of bronchiolitis in infants. Ital. J. Pediatr..

[16] O’Brien S., Borland M.L., Cotterell E., Armstrong D., Babl F., Bauert P., Brabyn C., Garside L., Haskell L., Levitt D. (2018). Australasian bronchiolitis guideline. J. Paediatr. Child Health.

[17] NICE (2020). Bronchiolitis in Children: Diagnosis and Management. London: National Institute for Health and Care Excellence (NICE); 2021 Aug 9. Should Probably be: National Institute for Health and Care Excellence. Bronchiolitis in Children: Diagnosis and Management|Guidance|NICE. https://www.nice.org.uk/guidance/ng9.

[18] Kirolos A., Manti S., Blacow R., Tse G., Wilson T., Lister M., Cunningham S., Campbell A., Nair H., Reeves R.M. (2020). A Systematic Review of Clinical Practice Guidelines for the Diagnosis and Management of Bronchiolitis. J. Infect. Dis..

[19] Friedman J.N., Rieder M.J., Walton J.M., Canadian Paediatric Society, Acute Care Committee, Drug Therapy and Hazardous Substances Committee (2014). Bronchiolitis: Recommendations for diagnosis, monitoring and management of children one to 24 months of age. Paediatr. Child Health.

[20] Verstraete M., Cros P., Gouin M., Oillic H., Bihouée T., Denoual H., Barzic A., Duigou A.L., Vrignaud B., Levieux K. (2014). Prise en charge de la bronchiolite aiguë du nourrisson de moins de 1 an: Actualisation et consensus médical au sein des hôpitaux universitaires du Grand Ouest (HUGO) [Update on the management of acute viral bronchiolitis: Proposed guidelines of Grand Ouest University Hospitals]. Arch. Pediatr..

[21] Manti S., Licari A., Brambilla I., Caffarelli C., Calvani M., Cardinale F., Ciprandi G., Cravidi C., Duse M., Martelli A. (2021). Agreements and controversies of national guidelines for bronchiolitis: Results from an Italian survey. Immun. Inflamm. Dis..

[22] Tang G., Lin J., Zhang Y., Shi Q. (2021). The effects and safety of continuous positive airway pressure in children with bronchiolitis: A systematic review and meta-analysis. J. Trop. Pediatr..

[23] Turnham H., Agbeko R.S., Furness J., Pappachan J., Sutcliffe A.G., Ramnarayan P. (2017). Non-invasive respiratory support for infants with bronchiolitis: A national survey of practice. BMC Pediatr..

[24] Bozzola E., Ciarlitto C., Guolo S., Brusco C., Cerone G., Antilici L., Schettini L., Piscitelli A.L., Chiara Vittucci A., Cutrera R. (2021). Respiratory Syncytial Virus Bronchiolitis in Infancy: The Acute Hospitalization Cost. Front. Pediatr..

[25] Loconsole D., Centrone F., Rizzo C., Caselli D., Orlandi A., Cardinale F., Serio C., Giordano P., Lassandro G., Milella L. (2022). Out-of-Season Epidemic of Respiratory Syncytial Virus during the COVID-19 Pandemic: The High Burden of Child Hospitalization in an Academic Hospital in Southern Italy in 2021. Children.

[26] Agüera M., Melé-Casas M., Molina M.M., Pons-Odena M., de-Sevilla M.F., García-García J.J., Launes C., Monfort L. (2022). Safety and effectiveness of bubble continuous positive airway pressure as respiratory support for bronchiolitis in a pediatric ward. Eur. J. Pediatr..

[27] Solana-Gracia R., Modesto i Alapont V., Bueso-Inchausti L., Luna-Arana M., Möller-Díez A., Medina A., Pérez-Moneo B. (2022). Changes in Ventilation Practices for Bronchiolitis in the Hospital Ward and Need for ICU Transfer over the Last Decade. J. Clin. Med..

[28] Ko M.S.M., Poh P.F., Heng K.Y.C., Sultana R., Murphy B., Ng R.W.L., Lee J.H. (2022). Assessment of Long-term Psychological Outcomes After Pediatric Intensive Care Unit Admission: A Systematic Review and Meta-analysis. JAMA Pediatr..

[29] Heikkilä P., Forma L., Korppi M. (2015). Hospitalisation costs for infant bronchiolitis are up to 20 times higher if intensive care is needed. Acta Paediatr..

[30] Øymar K., Bårdsen K. (2014). Continuous positive airway pressure for bronchiolitis in a general paediatric ward; a feasibility study. BMC Pediatr..

[31] Paredes González E., Campaña M.B., Salomón Moreno B., Rupérez Lucas M., de la Morena Martínez R. (2019). Non-invasive ventilation in acute bronchiolitis on the ward. A viable option. An. Pediatr..

[32] Van Brusselen D., De Troeyer K., Ter Haar E., Vander Auwera A., Poschet K., Van Nuijs S., Bael A., Stobbelaar K., Verhulst S., Van Herendael B. (2021). Bronchiolitis in COVID-19 times: A nearly absent disease?. Eur. J. Pediatr..

[33] Guedj R., Lorrot M., Lecarpentier T., Leger P.L., Corvol H., Carbajal R. (2021). Infant bronchiolitis dramatically reduced during the second French COVID-19 outbreak. Acta Paediatr..

[34] Cohen R., Ashman M., Taha M.K., Varon E., Angoulvant F., Levy C., Rybak A., Ouldali N., Guiso N., Grimprel E. (2021). Pediatric Infectious Disease Group (GPIP) position paper on the immune debt of the COVID-19 pandemic in childhood, how can we fill the immunity gap?. Infect. Dis. Now.

[35] Nolasco S., Manti S., Leonardi S., Vancheri C., Spicuzza L. (2022). High-Flow Nasal Cannula Oxygen Therapy: Physiological Mechanisms and Clinical Applications in Children. Front. Med..

[36] Fainardi V., Abelli L., Muscarà M., Pisi G., Principi N., Esposito S. (2021). Update on the Role of High-Flow Nasal Cannula in Infants with Bronchiolitis. Children.

[37] Tortosa F., Izcovich A., Carrasco G., Varone G., Haluska P., Sanguine V. (2021). Highflow oxygen nasal cannula for treating acute bronchiolitis in infants: A systematic review and meta-analysis. Medwave.

[38] Willer R.J., Brady P.W., Tyler A.N., Treasure J.D., Coon E.R. (2023). The Current State of High-Flow Nasal Cannula Protocols at Children’s Hospitals. Hosp. Pediatr..

[39] O’Brien S.L., Haskell L., Tavender E.J., Wilson S., Borland M.L., Oakley E., Dalziel S.R., Gill F.J. (2023). Factors influencing health professionals’ use of high-flow nasal cannula therapy for infants with bronchiolitis—A qualitative study. Front. Pediatr..

[40] Dafydd C., Saunders B.J., Kotecha S.J., Edwards M.O. (2021). Efficacy and safety of high flow nasal oxygen for children with bronchiolitis: Systematic review and meta-analysis. BMJ Open Respir. Res..

[41] Moreno M.G., del Villar Guerra P., Medina A., I Alapont V.M., Bournissen L.C., Veiga A.M., Ochoa-Sangrador C. (2023). High-Flow Oxygen and Other Noninvasive Respiratory Support Therapies in Bronchiolitis: Systematic Review and Network Meta-Analyses. Pediatr. Crit. Care Med..

[42] O’Brien S., Haskell L., Schembri R., Gill F.J., Wilson S., Borland M.L., Oakley E., Dalziel S.R., the Paediatric Research in Emergency Departments International Collaborative (PREDICT) network, Australasia (2022). Prevalence of high flow nasal cannula therapy use for management of infants with bronchiolitis in Australia and New Zealand. J. Paediatr. Child Health.

[43] Lin J., Zhang Y., Xiong L., Liu S., Gong C., Dai J. (2019). High-flow nasal cannula therapy for children with bronchiolitis: A systematic review and meta-analysis. Arch. Dis. Child..

[44] Franklin D., Babl F.E., George S., Oakley E., Borland M.L., Neutze J., Acworth J., Craig S., Jones M., Gannon B. (2023). Effect of Early High-Flow Nasal Oxygen vs. Standard Oxygen Therapy on Length of Hospital Stay in Hospitalized Children with Acute Hypoxemic Respiratory Failure: The PARIS-2 Randomized Clinical Trial. JAMA.

[45] Kooiman L., Blankespoor F., Hofman R., Kamps A., Gorissen M., Vaessen-Verberne A., Heuts I., Bekhof J. (2023). High-flow oxygen therapy in moderate to severe bronchiolitis: A randomised controlled trial. Arch. Dis. Child..

[46] Treasure J.D., Lipshaw M.J., Dean P., Paff Z., Arnsperger A., Meyer J., Gillen M., Segev N., Woeste L., Mullaney R. (2023). Quality Improvement to Reduce High-Flow Nasal Cannula Overuse in Children with Bronchiolitis. Pediatrics.

[47] Jat K.R., Dsouza J.M., Mathew J.L. (2022). Continuous positive airway pressure (CPAP) for acute bronchiolitis in children. Cochrane Database Syst. Rev..

